# Contribution of membrane-associated oscillators to biological timing at different timescales

**DOI:** 10.3389/fphys.2023.1243455

**Published:** 2024-01-09

**Authors:** Monika Stengl, Anna C. Schneider

**Affiliations:** Department of Biology, Animal Physiology/Neuroethology, University of Kassel, Kassel, Germany

**Keywords:** endogenous clocks, coupled oscillators, homeostasis, plasticity, circadian rhythms, ultradian rhythms, TTFL, PTFL

## Abstract

Environmental rhythms such as the daily light-dark cycle selected for endogenous clocks. These clocks predict regular environmental changes and provide the basis for well-timed adaptive homeostasis in physiology and behavior of organisms. Endogenous clocks are oscillators that are based on positive feedforward and negative feedback loops. They generate stable rhythms even under constant conditions. Since even weak interactions between oscillators allow for autonomous synchronization, coupling/synchronization of oscillators provides the basis of self-organized physiological timing. Amongst the most thoroughly researched clocks are the endogenous circadian clock neurons in mammals and insects. They comprise nuclear clockworks of transcriptional/translational feedback loops (TTFL) that generate ∼24 h rhythms in clock gene expression entrained to the environmental day-night cycle. It is generally assumed that this TTFL clockwork drives all circadian oscillations within and between clock cells, being the basis of any circadian rhythm in physiology and behavior of organisms. Instead of the current gene-based hierarchical clock model we provide here a systems view of timing. We suggest that a coupled system of autonomous TTFL and posttranslational feedback loop (PTFL) oscillators/clocks that run at multiple timescales governs adaptive, dynamic homeostasis of physiology and behavior. We focus on mammalian and insect neurons as endogenous oscillators at multiple timescales. We suggest that neuronal plasma membrane-associated signalosomes constitute specific autonomous PTFL clocks that generate localized but interlinked oscillations of membrane potential and intracellular messengers with specific endogenous frequencies. In each clock neuron multiscale interactions of TTFL and PTFL oscillators/clocks form a temporally structured oscillatory network with a common complex frequency-band comprising superimposed multiscale oscillations. Coupling between oscillator/clock neurons provides the next level of complexity of an oscillatory network. This systemic dynamic network of molecular and cellular oscillators/clocks is suggested to form the basis of any physiological homeostasis that cycles through dynamic homeostatic setpoints with a characteristic frequency-band as hallmark. We propose that mechanisms of homeostatic plasticity maintain the stability of these dynamic setpoints, whereas Hebbian plasticity enables switching between setpoints via coupling factors, like biogenic amines and/or neuropeptides. They reprogram the network to a new common frequency, a new dynamic setpoint. Our novel hypothesis is up for experimental challenge.

## 1 Introduction

### 1.1 Multiscale environmental rhythms: zeitgebers for multiscale clocks

Life on Earth evolved in a highly rhythmic environment. Geophysical rhythms occur via cycles of Earth-Moon-Sun constellations and additional rhythms are generated by multiscale fast signaling between organisms. These environmental rhythms favored the evolution of multiscale endogenous clocks that allow organisms to predict relevant changes in environmental parameters, improving adaptation and survival (Pittendrigh, 1993; Franken et al., 1994; Kippert and Hunt, 2000; Tyson et al., 2003; Rodríguez-Sosa et al., 2008; Isomura and Kageyama, 2014; Takahashi, 2016; Aviram et al., 2021). Any rhythmically occurring external cue can function as a zeitgeber when it entrains an endogenous clock (Meijer and Schwartz, 2003; Yoshii et al., 2015). The entrained clock maintains the same frequency and a stable phase-relation to the zeitgeber’s rhythm. If an organism’s clock expresses specific receptors, like photo- or chemoreceptors, it can entrain to zeitgeber cues of different modalities (Meijer and Schwartz, 2003; Stengl and Schröder, 2021). Predominant zeitgebers are the daily rhythm of light and dark, circalunar monthly rhythms in the brightness of nocturnal light, and infradian (period >24 h) annual rhythms in the duration of light per day (photoperiod). Consequently, environmental rhythms of light, often associated with regular temperature rhythms, temporally structure life on our planet into days, months, and years.

Superimposed on slow rhythms in illumination are fast fluctuating environmental events that organisms need to detect and process. Ultradian rhythms of social signals, most of them chemosensory, are exchanged between individuals within and across species, with periods ranging from milliseconds to hours (Stengl and Schröder, 2021). Concentration changes in nutritious or hazardous chemicals provide spatio-temporal orientation to food sources or mates, or away from danger. Chemoreceptors detect fast fluctuations in chemicals (e.g., in turbulent water or air) that occur on the scale of milliseconds (Baker et al., 1985; Stengl, 2010; Stengl and Schröder, 2021). These fast ultradian odor fluctuations are superimposed on slow 24 h cycles of odor presence; for example, plants advertise their nectar to attract pollinating insects only at specific times during the day, in coordination with daily patterned release of pheromones by mate-calling insects (Itagaki and Conner, 1988; Fenske and Imaizumi, 2016; Fenske et al., 2018). These superimposed oscillations in the concentration of species-specific chemicals can act as multiscale zeitgebers coordinating multiscale endogenous clocks that orchestrate physiology and behavior. Thus, endogenous clocks allow for ultradian (period <24 h), infradian (>24 h), or circadian (∼24 h) orchestration of reproduction rates (Hunt and Sassone-Corsi, 2007; Johnson, 2010; Farshadi et al., 2020), coordinate circadian rest-activity (sleep-wake) rhythms, as well as circannual adaptations to seasons in single cell organisms, animals, and humans (Meijer and Schwartz, 2003; Yoshii et al., 2015; Helfrich-Förster, 2018; Häfker and Tessmar-Raible, 2020; Veedin Rajan et al., 2021).

In summary, endogenous clocks predict environmental rhythms at multiple timescales as imminent advantage for survival by providing cues for spatial-temporal orientation. The environmental niche of an organism determines the pace and phase of its clocks. Amongst different environmental zeitgebers the daily 24 h cycle of light and dark provides the most dominant timing cue for terrestrial life and was the driving force for the evolution of circadian clocks that control sleep-wake cycles (Aréchiga et al., 1993; Hall and Rosbash, 1993; Takahashi, 2016).

### 1.2 The focus of our review

In insects and mammals, physiological and behavioral processes cycle at different periods, e.g., locomotion, feeding, breathing, or heartbeat. They are coupled at various strengths and are temporally orchestrated by the brain’s neuronal clocks (King and Sehgal, 2020; Patel and Rangan, 2021; Parviainen et al., 2022; Ijspeert and Daley, 2023; Luhmann, 2023; Nakamura et al., 2023; Park et al., 2023). Current research in chronobiology focuses on circadian clocks and transcriptional/translational feedback loop (TTFL)-based clock(work)s in circadian genetic model organisms such as fruit flies and mice. The predominant view of biological timing is hierarchical. It interprets the network of circadian clock neurons that are hubs of photic entrainment as master circadian clock centers: the suprachiasmatic nucleus (SCN) of mammals, the pineal of some avian species, and the accessory medulla (AME) of insects (Stetson and Watson-Whitmyre, 1976; Inouye and Kawamura, 1979; Takahashi and Menaker, 1979; Moore, 1983; Reischig and Stengl, 2003b; Reischig and Stengl, 2003a). Furthermore, the clock neuron’s TTFL clockwork is assumed to constitute the molecular master clock that drives all circadian oscillations of the clock cell as master clockwork outputs (Hardin, 2011; Harvey et al., 2020; Patton and Hastings, 2023).

Instead, here, we advocate a systems view of mutually interconnected endogenous TTFL and posttranslational feedback loop (PTFL) oscillators/clocks in single clock cells which run at multiple timescales. Furthermore, we suggest that clock neurons in the brain maintain a dynamically coupled and interconnected system of timing where single clock neurons can be recruited into different physiological/behavioral tasks, depending on coupling factors and zeitgebers. We do not attempt to provide a comprehensive review on timing at the levels of networks, transcription, translation, or metabolomics and refer to other reviews (e.g., Colwell, 2011; Hardin, 2011; Panda, 2016; Harvey et al., 2020; Parnell et al., 2021; Liu and Chiu, 2022; Adlanmerini and Lazar, 2023; Kahn et al., 2023; Patton and Hastings, 2023; Xiong and Garfinkel, 2023).

Our focus is the neuronal plasma membrane of mammalian and insect circadian clock neurons as a prominent endogenous PTFL clock ticking at multiple, superimposed timescales. First, we explain general properties of endogenous oscillators (Section 1.3). Then, we dive into details to elucidate also for the non-electrophysiologist how spontaneous ultradian membrane potential oscillations in clock neurons arise (Section 1.4) as mandatory prerequisite to circadian or any other frequency modulation. Briefly, important connections between membrane potential oscillations and second messenger oscillations at membrane associated signalosomes are pointed out before we sketch the neurophysiological concepts of neuronal homeostasis and plasticity, referring to more extensive reviews (Marder et al., 1996; Turrigiano, 1999; Turrigiano, 2008; Turrigiano, 2012; Turrigiano and Nelson, 2000; Caporale and Dan, 2008; Masquelier et al., 2009; Schulz and Lane, 2017; Lee and Kirkwood, 2019; McCormick et al., 2020; Debanne and Inglebert, 2023; Xiong and Garfinkel, 2023). In Section 2 we review the predominant hierarchical view on circadian clocks and contrast it with our novel systemic hypothesis of biological timing. In Section 3 we propose that mechanisms of homeostatic plasticity based on signalosomes maintain the stability of dynamic physiological setpoints in the system of interconnected PTFL and TTFL oscillators/clocks. Finally, in Section 4 we present our new systemic hypothesis of the neuronal plasma membrane as endogenous PTFL clock that is ticking at multiple timescales and interconnected with the endogenous TTFL clock as basis for a new concept of a dynamic homeostasis in physiology and behavior.

### 1.3 General properties of endogenous oscillators and clocks

**FIGURE 1:**
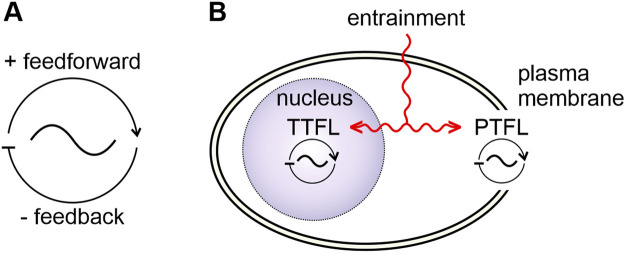
Basic properties of biological oscillators and clocks. **(A)** Biological oscillators consist of a positive feedforward- and a delayed negative feedback loop. **(B)** A clock is a specialized oscillator with entrainment mechanisms (red) that allow for synchronization to zeitgeber rhythms. Two general types of oscillators/clocks can be distinguished according to their “clockwork”: a transcription/translation feedback loop (TTFL)- based oscillator/clock, located in the nucleus (purple) and an oscillator/clock based on posttranslational feedback loops (PTFLs) located in other parts of the cell, such as the plasma membrane. Zeitgeber signals can entrain both the TTFL and the PTFL clockworks.

In organisms, endogenous oscillators generate self-sustained periodic events with stable cycle frequencies, even in the absence of rhythmic inputs (Figure 1). In the study of dynamical systems they are described mathematically as limit cycle oscillators with the limit cycle as the stable frequency (an attractor, here coined as “dynamic setpoint”) the oscillator returns to after perturbations (Leloup et al., 1999). Endogenous oscillators employ universal mechanisms despite their large variety, ranging from cycling conformations of molecular complexes to cells with oscillating membrane potentials to synchronously oscillating neural networks that orchestrate rhythmic behavior (Trujillo et al., 2019; Hille, 2022; Jabbur and Johnson, 2022; Fernandez-Ruiz et al., 2023; Kang et al., 2023). Oscillations are generated as soon as antagonistic elements/chemical reactions co-evolved (Gutekunst, 2018) and assembled to form a loop: positive feedforward pathways that are connected to delayed self-inhibitory feedback loops (Figure 1A). For example, genetic and molecular oscillators can be based on autoregulatory TTFLs (Figure 1B) (Hardin, 2011; Li et al., 2023b; Patton and Hastings, 2023). In contrast, not all feedback loops require transcription and translation. Post-translational feedback loops (PTFL, also known as post-translational oscillator: PTO; Figure 1B) work, for example, through cycles of autophosphorylation of molecular complexes. Positive feedforward elements promote autophosphorylation, while antagonistic negative feedback elements inhibit autophosphorylation (Bell-Pedersen et al., 2005; Johnson, 2010; Jabbur and Johnson, 2022; Li et al., 2023b).

**FIGURE 2:**
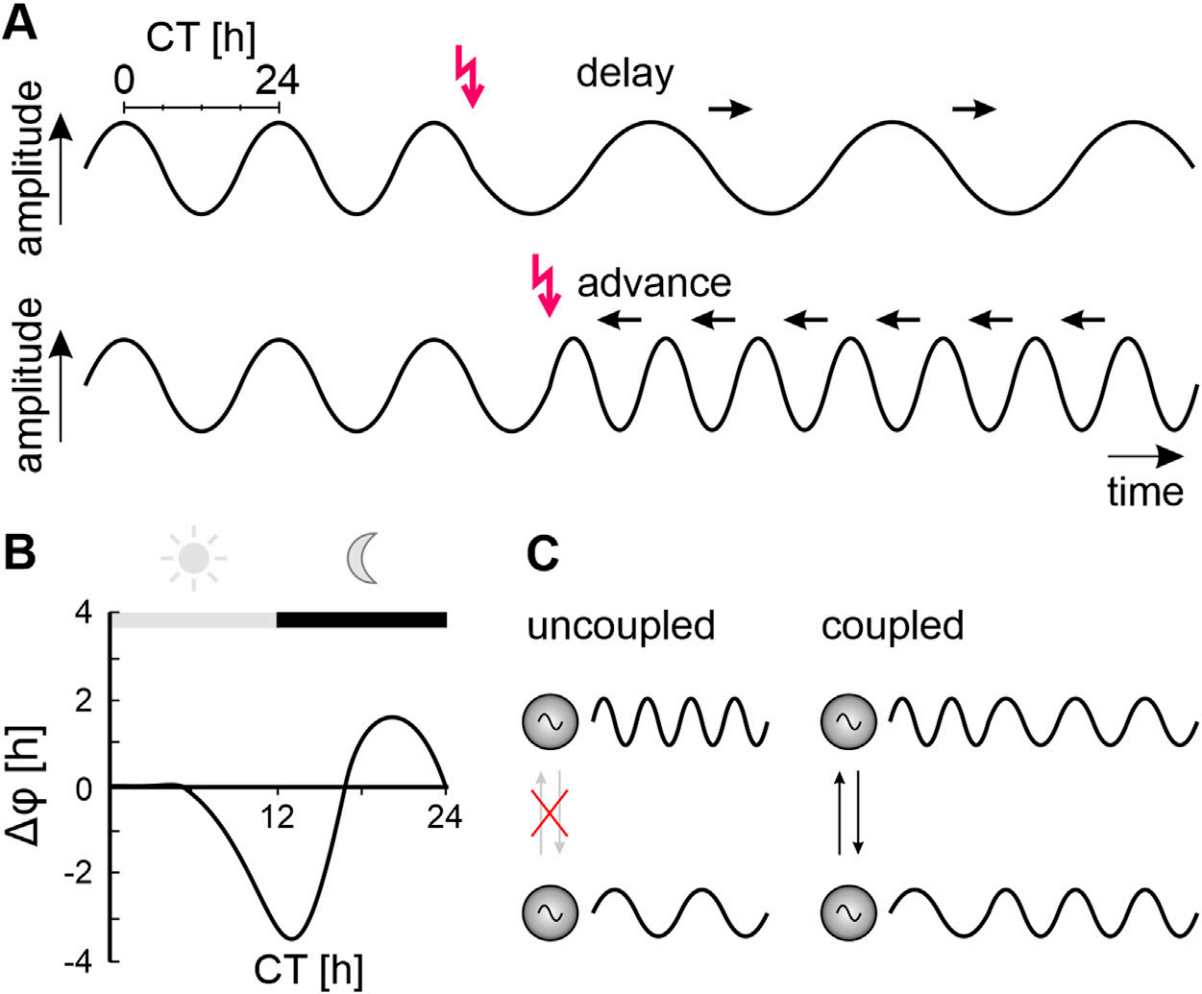
Phase-response curves characterize oscillators and clocks. **(A)** Inputs (red arrows) to an endogenous oscillator (sine waves) can either delay (top panel) or advance (bottom panel) the beginning of the next cycle, depending on the phase (relative timepoint within an endogeous cycle) at which they occur. The endogenous period (duration of the endogenous oscillator’s cycle) is measured in constant conditions. It is the shortest time difference between the respective same phases of the cycle divided into 24-time units (circadian time, CT). Repetitively delaying inputs (black arrows, top panel) prolong the period of the cycle (decrease oscillator frequency). Repetitively advancing inputs (black arrows, bottom panel) shorten the period of the cycle (increase oscillator frequency). **(B)** Plotting phase shifts against the circadian time (CT) of a subjective day-night cycle in constant conditions at which the phase-shifting input occurred yields a phase-response curve (PRC). Schematic PRC obtained from a nocturnal cockroach in a running wheel in constant darkness with light pulses applied at different CTs. Light pulses during the early subjective day (light grey bar, grey sun) have no effect. Light at the beginning of the subjective night (black bar, grey moon) delays the onset of locomotor activity rhythms, while light at the end of the night advances their onset. **(C)** Autonomous synchronization of two previously not interacting (crossed-out grey arrows) oscillators with endogenous fast (upper sine wave) or endogenous slow oscillations (lower sine wave). Mutual phase shifting through coupling/interactions (black arrows) results in a self-organized common period and stable phase relationship between both oscillators.

Interaction between endogenous oscillators with similar periods cause autonomous synchronization (Figure 2). This is the foundation for stable, self-organized timing, for autonomous assembly of a highly ordered sustainable biological system. Autonomous synchronization happens because inputs into an oscillator do not cause runaway acceleration or runaway braking but have phase-dependent antagonistic effects (Figures 2A, B). Self-organized, autonomous synchronization occurs because coupled oscillators either advance/accelerate or delay/decelerate each other until they maintain a common stable, intermediate frequency (a new dynamic setpoint) at stable phase relationships (Figure 2) (Tokuda et al., 2015; 2020; Ananthasubramaniam et al., 2018; Hahn et al., 2019; Lowet et al., 2022). Dependent on the context some oscillators become dominant and impose their frequency on other oscillators. While these pacemakers dictate the period of an oscillatory system, the coupled follower components can further pattern the system’s output (Marder and Bucher, 2001; Harris-Warrick, 2010; Daur et al., 2016; Marder et al., 2017; Jékely et al., 2018).

The term “clock” is less well defined than the term “oscillator” (Pittendrigh, 1993; Hall, 2003; 2005; Michel and Meijer, 2020). Generally, a clock is defined as a specialized endogenous oscillator that can be entrained by environmental zeitgebers. For example, a light-sensitive clock synchronizes (entrains) to the daily light-dark cycle. Entrainment by the zeitgeber via iterative phase-dependent phase-shifts eventually synchronizes and phase-locks the endogenous clock to the zeitgeber (Figures 2A, B). Because the entrained endogenous circadian clock has now, for example, the same 24 h period and a stable phase relationship to the rising and setting Sun, it can predict time of day. The endogenous period of clocks is species-specific and genetically determined and maintains stable oscillations even under constant conditions. In addition to unilateral entrainment by a zeitgeber the mutual coupling of endogenous clocks (Figure 2C) allows for autonomous multilateral synchronization which adds dynamics on a higher level than unilateral entrainment (Hardin, 2011; Hahn et al., 2019; Tokuda et al., 2020; Lowet et al., 2022; Jagannath et al., 2023; Kageyama et al., 2023; Kahn et al., 2023; Patton and Hastings, 2023; Wollmuth and Angert, 2023).

Endogenous clocks have a biochemical basis. While an increase in temperature usually speeds up biochemical processes, circadian clocks are temperature-compensated (being stable at different temperatures). Their endogenous cycle period remains the same over changing temperatures within a physiological range. Temperature compensation occurs automatically if both the positive feedforward and the negative feedback elements are symmetrically affected by temperature changes. Nevertheless, temperature changes can phase shift circadian clocks, for example, if they target only one of the clocks’ antagonistic elements (Rensing and Ruoff, 2002; Narasimamurthy and Virshup, 2017; Giesecke et al., 2023). So far, temperature-compensation is generally not considered a requirement for the definition of fast ultra- or slow infradian clocks, such as cell cycle clocks, metabolic clocks, photoperiodic clocks, or the clock that determines life span in a population of unicellular organisms (Rodríguez-Sosa et al., 2008; Zhu et al., 2017; Droin et al., 2019). Only few studies reported temperature-compensation for ultradian oscillators (Curras and Boulant, 1989; Roemschied et al., 2014; Städele et al., 2015; O’Leary and Marder, 2016).

In summary, endogenous oscillators evolved at different levels of complexity and timescales when antagonistic elements coupled to form robustly oscillating feedback loops. Respective endogenous oscillations are not an unwanted artefact but a process that can couple and reconcile antagonistic mechanisms into a stable system. Autonomous synchronization/entrainment of endogenous oscillators underlies the autonomously generated robust sustainable order and homeostasis of biological systems and embed organisms into their environmental niche (Schulz and Lane, 2017; Hahn et al., 2019; Liang et al., 2019; Rojas et al., 2019; Trujillo et al., 2019; Lowet et al., 2022; Jagannath et al., 2023; Kageyama et al., 2023; Kahn et al., 2023; Patton and Hastings, 2023; Wollmuth and Angert, 2023). The stable limit cycle oscillation frequency of a coupled system of endogenous oscillators can be viewed as dynamic setpoint of physiological homeostasis that the system bounces back to after perturbations.

### 1.4 General mechanisms underlying endogenous oscillations in neuronal electrical activity linked to intracellular messenger cascades at different timescales provide for homeostasis and plasticity

So far, it is unknown how synchronization of endogenous oscillators/clocks can occur across largely different timescales. Here, we choose mammalian and insect brain neurons as an example to explain multiscale rhythms originating from the neuron’s excitable membrane.

Neurons evolved as neurosecretory cells destined for orchestration of internal physiology and for communication between organisms and environment (Colgren and Burkhardt, 2022; Burkhardt et al., 2023). Across species, neuron-like cells and neuronal circuits employ oscillation-based mechanisms to autonomously regulate and stabilize physiology and behavior, providing for homeostasis (Schulz and Lane, 2017; Li et al., 2023c; Hürkey et al., 2023; Nikitin et al., 2023; Norekian and Moroz, 2023; Stöber et al., 2023). Homeostasis is defined as a dynamic equilibrium state of an open dynamical system, of the self-regulation of organisms to maintain stability while remaining adaptive (Encyclopedia Britannica, 2023). Despite being under intense investigation the functional basis of these different autonomous types of adaptive neuronal oscillations is still not understood but on the cellular level they all rely on membrane potential oscillations.

#### 1.4.1 Establishing a neuronal membrane potential

Neurons, as any other cell type, are enclosed by semi-permeable membranes separating water-based ionic solutions with different osmotic values (Hille, 2001). Thus, across semi-permeable neuronal membranes two opposing, counter-balancing driving forces build up: an electric gradient and an osmotic gradient. Usually, neurons face extracellular solutions with K^+^ concentrations being ∼10 times lower than the intracellular K^+^ concentration and extracellular Na^+^ concentrations being ∼10 times higher than intracellular (Hille, 2001; Cohen et al., 2009; Wells et al., 2012; Mulet et al., 2023). Excitable membranes of neurons of different species express highly conserved voltage-dependent, specific, or unspecific, cation and anion channels (Hille, 2022). In the plasma membrane of un-stimulated, resting neurons specific K^+^ channels (two-pore K^+^, K_2P_) are constitutively open and create “leak” currents (Goldstein et al., 2001; Patel and Honoré, 2001; Talley et al., 2001). Driven by their osmotic gradient, K^+^ ions diffuse through K_2P_ channels out of the cell until osmotic and electrical gradients are at equilibrium. The resulting membrane potential at which no net flow of ions through the membrane’s ion channels occurs is termed equilibrium (or reversal) potential. The reversal potential for each type of ion channel depends on the intra- and extracellular concentrations of permeating ions (Hille, 2022). For K^+^ it is typically around −80 mV and for Na^+^ around +50 mV. The steepest chemical gradient and, thus, the strongest driving force across the neuronal membrane is formed by Ca^2+^ ions, with extracellular concentrations in the millimolar range and intracellular concentrations several orders of magnitude lower in the nano- to picomolar range. The ionic gradients across the membrane are maintained by energy-consuming electrogenic pumps (Läuger, 1991). Thus, neurons at rest remain stable at their negative K^+^ equilibrium potential because only leaky K_2P_ channels are open. Nevertheless, although K_2P_ channels usually do not show voltage dependence, they are tightly controlled by second messenger systems linking membrane potential to second messenger signaling as basis for homeostatic feedback control of neuronal activity (Goldstein et al., 2001; Patel and Honoré, 2001; Talley et al., 2001).

#### 1.4.2 Pacemaker channels are crucial for endogenous membrane potential oscillations

**FIGURE 3:**
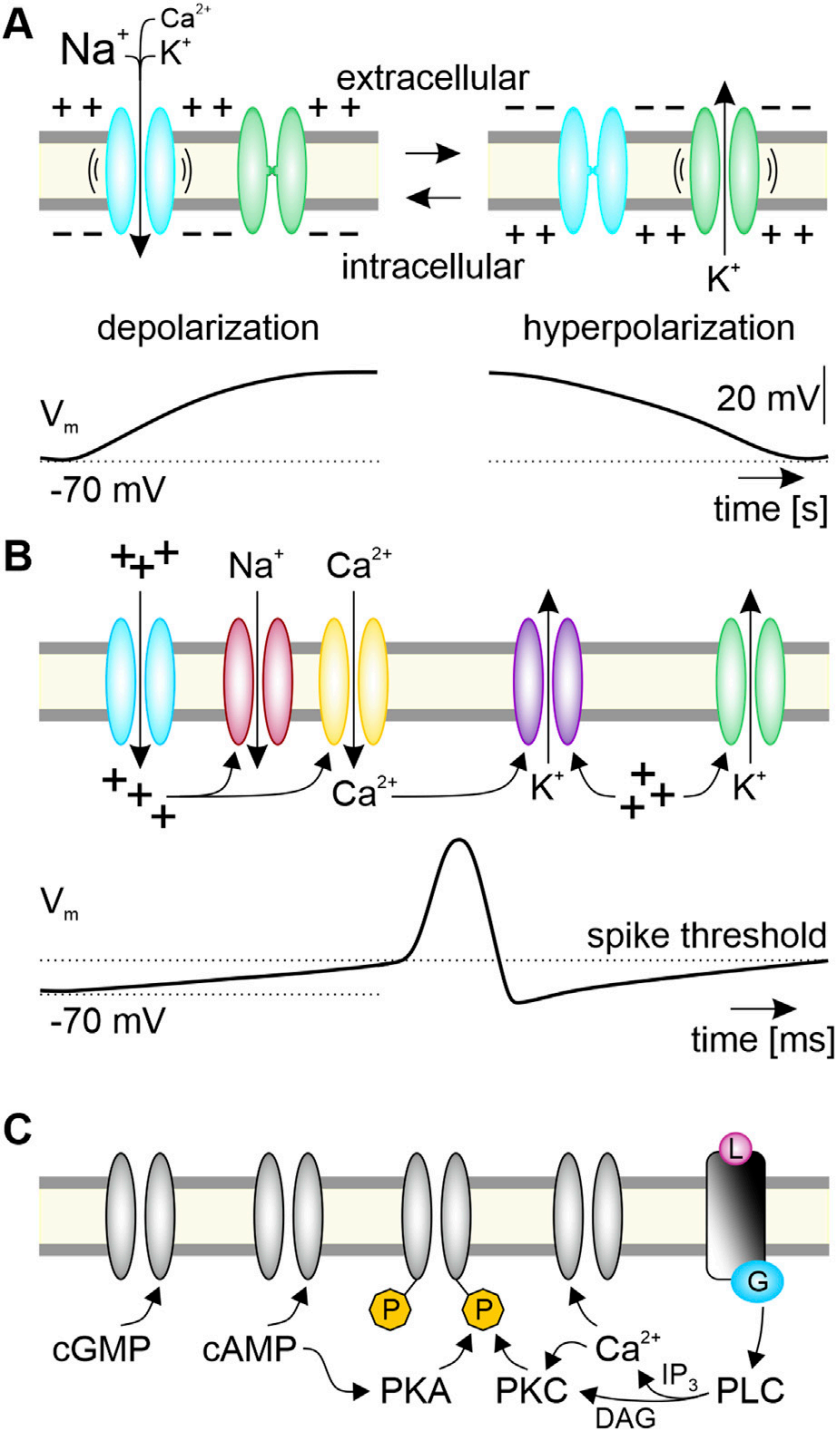
Ion channels contributing to endogenous oscillations of membrane potential and second messenger levels constitute a PTFL clock associated with a clock neuron’s plasma membrane. **(A)** Minimal requirement to generate spontaneous membrane potential oscillations are one pacemaker channel and one channel with antagonistic effects on the membrane potential. As example, the slow HCN-type pacemaker channels (blue) open at hyperpolarized voltages (“-”). The resulting influx of Na^+^, Ca^2+^, and K^+^ depolarizes the neuron from its negative membrane potential (−70 mV). This depolarization (“+”) opens voltage gated K^+^ channels (green), and the resulting efflux of K^+^ hyperpolarizes the neuron so that HCN channels can open again. Opening of hyperpolarization-activated HCN pacemaker channels constitute the positive feedforward element. The delayed negative feedback element, restarting the cycle of this posttranslational feedback loop (PTFL)-clock in the plasma membrane, is the slow HCN-dependent depolarization (“+”). It increases the open-time probability of the hyperpolarizing K^+^ channels and it closes the inverse-voltage-dependent HCN-pacemaker channels. **(B)** The respective complement of ion channels in a clock neuron leads to endogenous voltage oscillations at specific ultradian timescales. Depolarization via pacemaker channels (blue) triggers fast spikes via opening of voltage-gated fast Na^+^ channels (red) at the spike threshold. In addition, depolarization opens high voltage-activated (HVA-type) Ca^2+^ channels (yellow), increasing intracellular Ca^2+^ levels. Both depolarization and Ca^2+^ increase gate further channels, such as Ca^2+^-dependent (purple) and voltage-dependent (green) hyperpolarizing K^+^ channels. **(C)** Various interacting intracellular messenger cascades further gate and modulate antagonistic ion channels and enzymes via posttranslational modifications which leads to multiscale oscillations of the membrane potential interlinked with intracellular messenger oscillations. Cyclic nucleotides (cGMP, cAMP) modulate enzymes such as kinases (protein kinase A; PKA; protein kinase C; PKC) directly, and ion channels either directly, or indirectly via phosphorylating (P, yellow) kinases. Rising levels of intracellular messengers like Ca^2+^, as well as ligand (L, pink)-dependently activated G protein (blue)-coupled receptors activate and/or inhibit ion channels and enzymes. For example, activated phospholipase C (PLC) generates the second messengers diacyl glycerol (DAG) and inositol trisphosphate (IP_3_), orchestrating Ca^2+^-dependent signaling cascades. Positive feedforward elements of the PTFL clock cause depolarizations, delayed negative feedback elements hyperpolarize.

Characteristic for clock neurons is their ability to generate endogenous oscillations in membrane potential and action potential (spike) frequency at characteristic timescales. These endogenous oscillations occur even in the absence of electrical stimulation and are maintained at dynamic setpoints through mechanisms of homeostatic plasticity (Miller and Selverston, 1982; Adams and Benson, 1985; Marder et al., 1996; Turrigiano, 1999; Turrigiano and Nelson, 2000; Lee and Kirkwood, 2019; McCormick et al., 2020). Even though endogenous membrane potential oscillations can occur at various timescales, they all require the expression of pacemaker channels (Figure 3A). In general, pacemaker channels are cation channels that open at hyperpolarized voltages. They drive the membrane potential to more depolarized potentials (Lüthi and McCormick, 1998; Robinson and Siegelbaum, 2003; Bose et al., 2014; Cochet-Bissuel et al., 2014; Das et al., 2016; Golowasch et al., 2017; Ratliff et al., 2021; Sharma et al., 2023). The resulting depolarization both closes the pacemaker channels and typically activates voltage-gated K^+^ channels, which in turn hyperpolarize the membrane potential. The hyperpolarization causes pacemaker channels to open again, the cycle begins anew and results in membrane potential oscillations (Figure 3A). One prominent example of a pacemaker channel is the hyperpolarization-activated, cyclic nucleotide-gated (HCN) non-specific cation channel that gives rise to the *I*_h_ current (Atkinson et al., 2011; Combe and Gasparini, 2021; Crunelli et al., 2023). The HCN channels express reversed voltage dependence, i.e., they close with depolarization and open with hyperpolarization. In that way they resemble molecular oscillators (Lee and MacKinnon, 2019). The depolarization by *I*_h_ and other regenerative pacemaker currents counteract the K^+^ leak, increase the neuron’s excitability, and promote oscillations (Cymbalyuk et al., 2002; Blethyn et al., 2006; Golowasch et al., 2017; Schneider et al., 2021; Sharma et al., 2023). When the oscillating depolarizations reach the activation threshold (approximately −40 mV) of voltage-gated fast Na^+^ channels (Figure 3B) the neuron generates spikes. However, already the subthreshold membrane potential oscillations control response threshold and response kinetics of neurons. These oscillations are determined by the kinetics, permeabilities, and relative numbers of antagonistic (depolarizing vs. hyperpolarizing) ion channels with their respective posttranslational modifications (Figures 3B, C) (Hille, 2022). Furthermore, the HCN channel is a hub for interacting intracellular messenger cascades providing for tight homeostatic control of spontaneous neuronal activity (Atkinson et al., 2011; Puri, 2020; Combe and Gasparini, 2021; Crunelli et al., 2023). We will focus next on Ca^2+^ dependent intracellular signaling and its close coupling to the membrane potential in a multitude of negative feedback circuits that provide for homeostasis of neuronal functions.

#### 1.4.3 Ca^2+^ links membrane potential oscillations to molecular signaling pathways contributing to homeostatic control

Any neuron expresses various sets of voltage-gated Ca^2+^ channels (Hille, 2022; Sharma et al., 2023). Therefore, both subthreshold membrane potential oscillations and regular spiking are accompanied by oscillatory changes in the intracellular Ca^2+^ concentration. Many enzymes, proteins, ion channels, and transcription factors are regulated by Ca^2+^ (Puri, 2020; Tokumitsu and Sakagami, 2022). Hence, the increase in intracellular Ca^2+^ concentration can both directly or indirectly activate and inactivate additional ion channels, which adds layers of complexity to the membrane potential oscillations (Figures 3B, C). Because prolonged high intracellular Ca^2+^ concentrations are highly toxic to neurons, intracellular Ca^2+^ levels are under tight autonomous homeostatic control (Duchen, 2000; Rizzuto et al., 2004; Sundararaj et al., 2021; Centeno et al., 2023; Nieto-Felipe et al., 2023).

Homeostatic mechanisms keep the intracellular Ca^2+^ concentrations tightly at nano- to picomolar levels as a dynamic setpoint via a multitude of interlinked negative feedback control circuits. When the intracellular Ca^2+^ concentrations rises, plasma membrane Ca^2+^ channels are either directly closed via the Ca^2+^-binding protein calmodulin, or their open time probability is decreased via protein kinase C-dependent phosphorylation (Hille, 2022; Sharma et al., 2023). Also, the number of ion channels and transporters located in the plasma membrane are controlled via a barrage of Ca^2+^-dependent homeostatic processes based on negative feedback (Lamothe and Zhang, 2016). Furthermore, elevated intracellular Ca^2+^ activates pumps that transport Ca^2+^ out of the neuron or into intracellular Ca^2+^ stores such as the endoplasmatic reticulum. This intracellular store contains Ca^2+^-conducting IP_3_ receptors and ryanodine-type ion channels which open in tight cooperation with plasma membrane Ca^2+^ channels when intracellular Ca^2+^ concentrations drop below the homeostatic setpoint and need to be restored (Duchen, 2000; Rizzuto et al., 2004; Sundararaj et al., 2021; Centeno et al., 2023; Nieto-Felipe et al., 2023).

#### 1.4.4 Neuronal homeostasis and plasticity are based on a system of coupled oscillators

Mechanisms of homeostatic plasticity constitute an interconnected network of feedforward/feedback elements. They are characteristic for endogenous oscillators and clocks that control neuronal activity at the single cell level as well as at the level of neuronal networks (Debanne and Inglebert, 2023; Xiong and Garfinkel, 2023). Traditionally, it is thought that homeostatic mechanisms evolved to maintain stationary setpoints of various physiological parameters such as fixed setpoints of electrical activity after stimulus-dependent activations (Marder et al., 1996; Turrigiano, 1999; Turrigiano and Nelson, 2000; Caporale and Dan, 2008; Masquelier et al., 2009; Schulz and Lane, 2017; Lee and Kirkwood, 2019; McCormick et al., 2020). Now, it is increasingly appreciated that homeostatic setpoints are not stationary but oscillating. In that way, they are “dynamic setpoints”. These observed physiological oscillations are not an undesired oversteering of feedback control circuits. Instead, oscillation-based coupling as basis for dynamic homeostatic setpoints allow for autonomously regulated plasticity. Oscillations interconnect and stabilize incompatible, antagonistic conditions for individual elements, such as oxidation and reduction, or phosphorylation and dephosphorylation (Xiong and Garfinkel, 2023).

On the single neuron level, mechanisms of homeostatic plasticity keep endogenous membrane potential oscillations at physiological dynamic setpoints, and maintain a characteristic spiking frequency via homeostatic control of ion channels (Xiong and Garfinkel, 2023). The negative feedback element of homeostatic control can be Ca^2+^ entry via the pacemaker channel that feeds back to decrease its open time probability, either directly or indirectly via phosphorylation by Ca^2+^-dependent protein kinase (He et al., 2014). Thereby, neuronal activity and responsiveness is oscillating but maintained within a stable physiological range.

**FIGURE 4:**
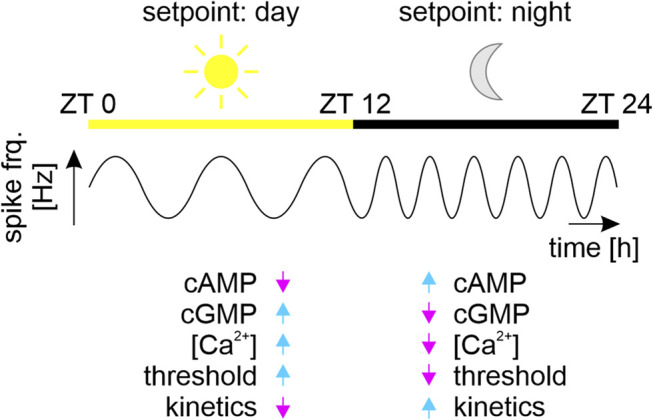
Different dynamic setpoints during the sleep-wake cycle. Schematic of synchronized physiological states of brain networks (ensembles) of clock neurons that regulate circadian sleep-wake cycles of a nocturnal animal. The multiscale clock neurons of the circadian ensembles fire at ultradian fast frequencies with superimposed circadian modulation. Ensembles fire with a common lower ultradian frequency (sine waves) during the day (sun, yellow bar, Zeitgebertime (ZT) 0–12 h), as compared to the night (moon, black bar, ZT 12–24 h). Each stable ultradian action potential frequency is the fingerprint (coined “setpoint”) of an ensemble of coupled oscillator neurons. The network’s “day” setpoint maintains the nocturnal animal’s physiological homeostasis of sleep, while the “night” setpoint maintains the physiological homeostasis of activity. Based on our work on peripheral insect circadian clock neurons we hypothesize that these antagonistic dynamic homeostatic setpoints correlate with antagonistic second messenger compositions (Stengl, 2010; Schendzielorz et al., 2012; Schendzielorz et al., 2015; Dolzer et al., 2021; Stengl and Schröder, 2021). The high sensory thresholds and slow response kinetics of sensory neurons during sleep correlate with increased intracellular Ca^2+^ and cGMP concentrations. In contrast, the low sensory thresholds and fast response kinetics during wakefulness correlate with increased cAMP levels and decreased concentrations in intracellular Ca^2+^ and cGMP levels (Stengl, 2010; Dolzer et al., 2021; Stengl and Schröder, 2021).

Furthermore, at the brain’s network level, homeostatic plasticity maintains a common, synchronized ultradian spiking frequency of ensembles of synchronized neurons as dynamic homeostatic ensemble setpoint by orchestrating the gain of all synapses in the neuronal network without changing their respective weights (Marder et al., 1996; Turrigiano, 1999; Turrigiano and Nelson, 2000; Schulz and Lane, 2017; Lee and Kirkwood, 2019; McCormick et al., 2020; Valakh et al., 2023). During the course of each day (i.e., with circadian modulation) mammalian brains pass through different self-organized stable oscillatory states (i.e., dynamic setpoints) via autonomous sequential recruitment of neuronal ensembles (Figure 4). These ensembles spike synchronously at evolutionary conserved ultradian spike frequency bands that are connected to specific physiological functions (Laurent et al., 2016; Singer and Lazar, 2016; Hahn et al., 2019; McCormick et al., 2020; Tononi and Cirelli, 2020; Cirelli and Tononi, 2022). Ultradian oscillations in neuronal ensemble activity with superimposed circadian modulation are, for example, slow delta (0.5–4 Hz) waves predominating during sleep, or fast gamma (40 to >100 Hz) waves predominating during wakefulness (Figure 4). They correlate with different physiological states of sensory perception, of learning and memory, of sleep or wakefulness (Buzsáki and Draguhn, 2004; Colwell, 2011; Harvey et al., 2020; Stengl and Schröder, 2021; Brodt et al., 2023; Patton and Hastings, 2023). As foundation of robust, autonomous neuronal functions multiple mechanisms of homeostatic plasticity at the level of single neurons as well as at the level of neuronal ensembles maintain a specific dynamic setpoint measurable as stable spike frequency (McCormick et al., 2020).

In contrast to homeostatic plasticity mechanisms that keep neuronal setpoints stable, mechanisms of Hebbian plasticity (including associative and non-associative forms of learning and memory), change neuronal setpoints. An example of non-associative learning is adaptation of olfactory sensory neurons. Olfactory adaptation employs, e.g., Ca^2+^-dependent negative feedback control mechanisms to prevent damage via overstimulation after a very strong or long odor/pheromone stimulus (Zufall and Leinders-Zufall, 2000; Dolzer et al., 2001; Spehr et al., 2009; Stengl, 2010). Hebbian plasticity mechanisms shift the current physiological dynamic setpoint to a new dynamic setpoint, for example, measurable as shifted dose-response curve of the sensory neurons. Thus, in an olfactory receptor neuron, after adapting to stimulation higher odor stimuli are necessary to further activate the sensory neuron which, thereby, preserved the memory of the adapting stimulus.

Hebbian plasticity at the network level allows for stimulus-dependent changes in brain function best described during synaptic processes of associative learning and memory (Hebb, 1949; Yee et al., 2017; Magee and Grienberger, 2020; McFarlan et al., 2023). In contrast to homeostatic plasticity Hebbian plasticity can push the neuronal network to a new physiological dynamic setpoint, detectable as different ensemble formation with different synchronous spiking frequency due to reconfiguration of the neuronal network. During processes of associative learning, such as long-term potentiation (LTP) or long-term depression (LTD), ultradian oscillatory processes in the pre- and postsynaptic cell are coupled at defined phase-differences to allow for spike time-dependent forms of plasticity causing synaptic weight changes (Debanne and Inglebert, 2023; Griffiths and Jensen, 2023; Yamakou et al., 2023). The autonomous synchronization of pre- and postsynaptic neurons to new synaptic weights, to a new physiological setpoint, depends on physiological conditions and specific coupling signals (neurotransmitters, neuropeptides) and can extend over several timescales (short, medium, long-term memory). Thus, mechanisms of homeostatic or Hebbian plasticity can also act on the circadian timescale and can target both the number of functional receptors as well as the gating and the number and types of voltage dependent ion channels expressed to regulate dynamic setpoints of electrical activity in neurons (Turrigiano et al., 1994; 1995; Marder et al., 1996; Golowasch et al., 1999; Turrigiano, 1999; Turrigiano and Nelson, 2000; Valakh et al., 2023).

In summary, evolutionarily conserved ultradian endogenous membrane potential oscillations at different timescales are a prerequisite to all endogenous (including circadian) clock neurons (Schneider and Stengl, 2005; Schneider and Stengl, 2006; Schneider and Stengl, 2007; Stengl and Schröder, 2021; Brodt et al., 2023). Spontaneously active neurons require the expression of pacemaker channels. Oscillations in membrane potential are tightly coupled to oscillations of intracellular Ca^2+^ levels. Both spike frequency and Ca^2+^ oscillations are controlled at dynamic setpoints via various interlinked mechanisms of homeostatic plasticity at multiple timescales. In contrast to mechanisms of homeostatic plasticity which preserve and maintain robust dynamic setpoints, mechanisms of Hebbian plasticity change the respective dynamic setpoint of the system of coupled oscillators/clocks (Turrigiano et al., 1994; Turrigiano et al., 1995; Marder et al., 1996; Golowasch et al., 1999; Turrigiano, 1999; Turrigiano and Nelson, 2000). It remains to be determined how concepts of endogenous clocks/oscillators are interconnected with, are the same as, or are different from concepts of homeostasis and plasticity in neurons.

## 2 Hierarchical versus systemic concepts of endogenous clocks

In the following sections, we briefly state general principles of the master molecular TTFL clockwork in insect and mammalian clock neurons. Subsequently, we focus on plasma membrane-derived rhythms in mammalian and insect circadian clock neurons to summarize prevailing concepts. Since mammalian neurons and brains are much better analyzed with electrophysiological methods, we focus more on the mammalian than the insect circadian system. We challenge the current hierarchical view that membrane-dependent circadian rhythms are mere outputs of the master TTFL clockwork without denying strong mutual coupling between both. We highlight open questions, taking a systemic view of interlinked oscillators that use mechanisms of homeostatic and Hebbian plasticity. Finally, we propose a new hypothesis how circadian and ultradian oscillations could be linked by mechanisms of neuronal plasticity involving an endogenous multiscale PTFL membrane clock.

### 2.1 Current standard model of an endogenous circadian clock neuron with a TTFL-based clockwork

The search for the molecular clockwork which underlies circadian rhythms in behavior was initiated by the finding of a single gene that affected daily rhythms of both eclosion and locomotion in the fruit fly *Drosophila melanogaster* (Konopka and Benzer, 1971). Since then, it was generally believed that a molecular genetic clockwork in the nucleus rules circadian behavior such as daily sleep-wake cycles.

Best studied are the molecular circadian TTFL-based neuronal clocks in insects and mammals that generate oscillations in mRNA and protein levels with periods of ∼24 h (Hall, 2003; Hall, 2005; Hardin, 2011; Hastings et al., 2019). The core mechanism of the TTFL clockwork is astoundingly conserved between animal species and consists of various sets of homologous circadian clock genes. The transcription factors CLOCK and CYCLE (mammalian ortholog: BMAL1) are the positive feedforward elements that activate the transcription of the circadian clock genes *period*, *timeless*, and/or *cryptochrome*s. Translation of mRNAs and posttranscriptional modifications of clock proteins (such as successive phosphorylations) generate delays before the clock proteins move back into the nucleus and inhibit their own transcription as negative feedback elements. This core circadian clockwork is interlinked with other oscillating TTFLs that increase stability and regularity of the resulting oscillations in mRNA and protein levels (Hardin, 2011; Mendoza-Viveros et al., 2017; Hastings et al., 2019; Park et al., 2020).

The prevailing assumption is that in mammalian or insect brains a single neuron becomes a circadian clock neuron because it contains this conserved TTFL-based molecular master clockwork that generates endogenous oscillations in gene transcription in the 24 h range. This TTFL master clockwork in master circadian clock neurons in the brain then drives all other circadian oscillations in this organism’s physiology and behavior, controlling and dominating peripheral clock cells in other organs (Green and Gillette, 1982; Groos and Hendriks, 1982; Welsh et al., 1995; Honma et al., 1998; Schaap et al., 2003; Colwell, 2011; Cox and Takahashi, 2019; Harvey et al., 2020; Patton and Hastings, 2023). Even though this review focuses on neuronal clocks in insects and mammals it should be noted that a wide variety of clock mechanisms, some of which are entirely independent of TTFLs, were reported in other organisms (Bell-Pedersen et al., 2005; Kitayama et al., 2008; Johnson, 2010; O’Neill and Reddy, 2011; Brown et al., 2012; Jabbur and Johnson, 2022; Li et al., 2023b). Furthermore, while different kinases, phosphatases, or O-GlcNAcylation processing enzymes regulate core elements of the neuron’s TTFL clockwork through posttranslational modifications (Tomita et al., 2005; Li et al., 2019; Anna and Kannan, 2021; Parnell et al., 2021; Liu and Chiu, 2022), it remains to be studied whether any of these signaling cascades constitute homeostatic control and/or are PTFL-based endogenous oscillators.

### 2.2 Plasma membrane-dependent oscillations are generally assumed to be outputs of the molecular master TTFL clockwork

Circadian clock gene expressing neurons in the brains of both insects and mammals can generate spontaneous membrane potential oscillations at fast and slow frequencies that elicit ultradian and circadian spike rhythms (Pennartz et al., 2002; Jackson et al., 2004; Brown and Piggins, 2007; Schneider and Stengl, 2007; Belle et al., 2009; Colwell, 2011; Allen et al., 2017; Belle and Allen, 2018; Harvey et al., 2020). In mammals, the suprachiasmatic nucleus (SCN) houses the circadian pacemaker network that controls sleep-wake cycles, as well as any other physiological and behavioral circadian rhythms (Meijer and Schwartz, 2003; Michel and Meijer, 2020; Patton and Hastings, 2023). Under physiological conditions, SCN clock neurons spike spontaneously with circadian rhythms and, additionally, with higher ultradian frequency during the day (theta: ∼4 Hz) than during the night (delta: ∼1 Hz). In wild-type (WT) rodents, circadian and ultradian spike rhythms persist in SCN slices in constant conditions (Green and Gillette, 1982; Groos and Hendriks, 1982) and in individually dispersed clock neurons of both invertebrates and mammals (Michel et al., 1993; Welsh et al., 1995; Honma et al., 1998; Michel and Meijer, 2020). Therefore, the generation of electrical activity rhythms in the ultradian and circadian range is not an exclusive property of the SCN’s neuronal network but a property of single neurons with endogenous circadian clockworks. However, synchronized electrical activity of the SCN clock neuron network is a prerequisite for robust daily rhythms in behavioral activity: Application of the Na^+^ channel blocker TTX to the SCN of rats reversibly blocked both ultradian and circadian rhythms in electrical activity that correlated with a reversible block of daily locomotor and drinking behavioral rhythms (Schwartz et al., 1987; Earnest et al., 1991; Honma et al., 1998; Schaap et al., 2003; Colwell, 2011; Harvey et al., 2020).

It is generally assumed that the TTFL-based molecular master clockwork in individual neurons generates the ∼24 h rhythms in the neuron’s membrane potential via transcriptional control of key ion channels (Depetris-Chauvin et al., 2011; Allen et al., 2017; Harvey et al., 2020). Indeed, if core clock genes such as *clock* of the mammalian core TTFL clockwork are mutated or deleted, circadian sleep-wake cycles become arrhythmic (Albus et al., 2002). In addition, explants of the SCN network of TTFL clockwork mutants express arrhythmic electrical activity. However, improved *clock* mutants with targeted removal of the exons encoding the required dimerization region for BMAL1 that led to a loss of CLOCK immunoreactivity still express robust circadian rhythms in locomotor activity with only slightly shorter periods, with altered light-responses, and with some milder alterations in the TTFL clockwork (Collins and Blau, 2006; DeBruyne et al., 2006). Individually dispersed SCN clock neurons of *clock* mutants still display endogenous spiking rhythms in the 24 h range, albeit with a wider range of periods compared to WT SCN neurons (Herzog et al., 1998). Mutations of circadian clock genes in brain areas outside the SCN affected but did not delete all circadian rhythms in these neuronal circuits (Ray et al., 2020; Bering et al., 2023; Zheng et al., 2023). Similarly, in *Drosophila*, the core clock genes are not necessarily required for rhythmicity, since arrhythmicity in *period* ^*01*^ mutants can be partially rescued by *cryptochrome* mutants (Collins et al., 2005). Therefore, the observed losses in rhythmicity in TTFL targeted mutants appear to be caused by loss of synchronization within and between neurons, rather than the loss of all endogenous circadian rhythmicity. Mutations in the core clock TTFL change the periodicity and phase of spiking and behavioral activity without abolishing rhythms (Wegner et al., 2017; Haque et al., 2019; Ray et al., 2020; Zheng et al., 2023). Thus, while there is convincing evidence for a tight coupling between membrane associated circadian oscillations and the circadian TTFL clockwork (Colwell, 2011; Harvey et al., 2020) it was not proven that all circadian membrane-associated rhythms are mere outputs of a master TTFL clock.

In summary, synchronized circadian rhythms in electrical activity of circadian clock neurons drive circadian rhythms in behavior. Mutations of core TTFL constituents are tightly coupled to, but do not delete all circadian membrane potential rhythms, possibly due to redundancy at the TTFL level, possibly due to other additional mechanisms such as endogenous PTFL oscillators/clocks. While nuclear clockwork and membrane potential rhythms clearly interact the respective elements and mechanisms are not yet known.

### 2.3 Silencing of neuronal activity affects the circadian TTFL clockwork

Circadian membrane potential oscillations feed back to the molecular TTFL clockwork (Mizrak et al., 2012; Harvey et al., 2020; Parnell et al., 2021; Matsuo et al., 2022). Electrically silencing or isolating circadian clock neurons of the SCN does not stop the molecular clockwork but renders its circadian rhythms less stable (Welsh et al., 1995; Herzog et al., 1998; Shirakawa et al., 2000; Yamaguchi et al., 2003; Aton et al., 2005; Lundkvist et al., 2005; Liu et al., 2007). In SCN clock neurons the blocking of Na^+^ channels and mutations of K^+^ ion channels (*Kcnc1/Kcnc2*) deleted circadian membrane potential rhythms without deleting circadian transcription of *per*2 of the core TTFL clockwork (Hermanstyne et al., 2023). Notably, in electrically silenced or isolated *Drosophila* neurons, transcription rhythms of the TTFL molecular clockwork desynchronized or dissipated altogether (Nitabach et al., 2002; Depetris-Chauvin et al., 2011; Schlichting et al., 2019; Tang et al., 2022). Apparently, the endogenous circadian molecular clockwork becomes more robust when it is tightly coupled to circadian spiking rhythms in the same clock neuron and/or the neuronal network.

**FIGURE 5:**
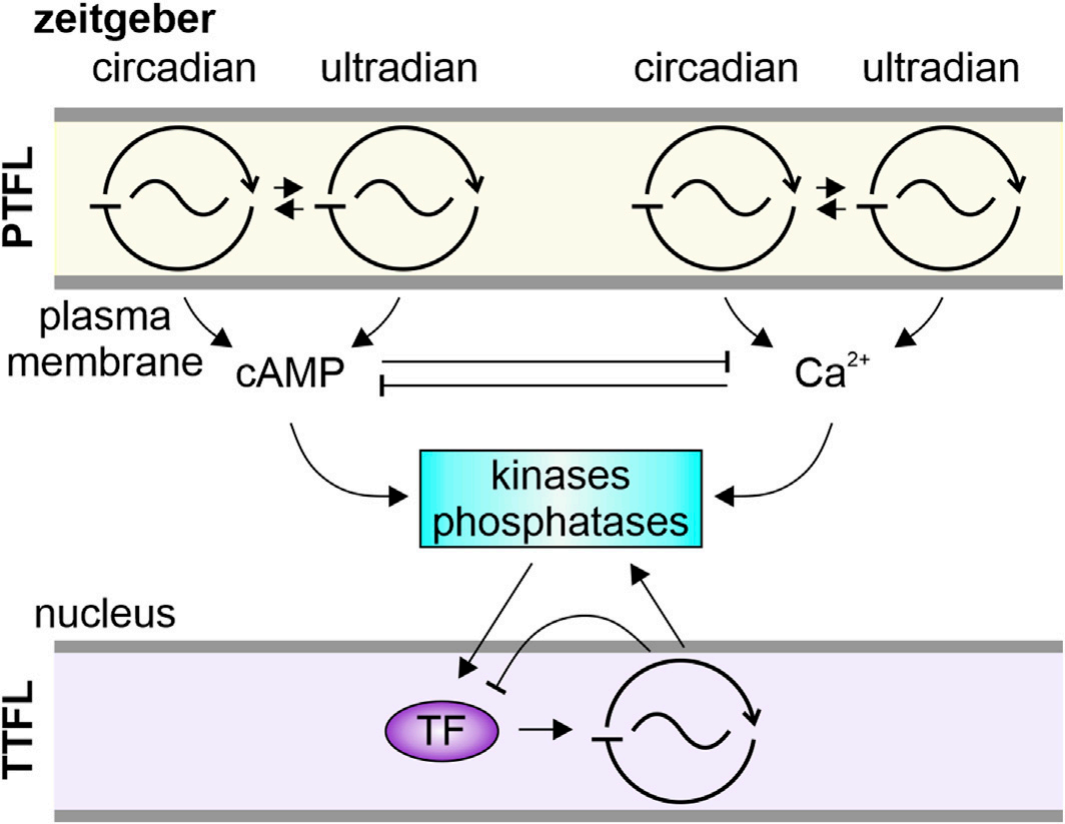
Schematic of our novel systemic hypothesis. Associated with the plasma membrane are multiple signalosomes comprising posttranslational feedback loop (PTFL)-oscillators. They anchor on scaffolding proteins pacemaker channels and specific receptors with their intracellular messenger cascades. Depending on the receptor, e.g., luminance- or chemoreceptor, the signalosome is susceptible to circadian or ultradian zeitgeber cues. Entrained to the zeitgeber frequency the signalosome’s transduction cascades generate oscillations in levels of cAMP and/or Ca^2+^. The intracellular messenger oscillations can interlink/couple by sharing common targets, like kinases and phosphatases. These can act as coupling factors synchronizing PTFL-oscillators as well as, via modulation of transcription factors (TF), transcriptional-translational feedback loop (TTFL) oscillators.

In general, in both insect and mammalian circadian clock neurons, the ultradian membrane potential rhythms that are tightly interlinked with circadian activity rhythms are also tightly coupled with intracellular circadian rhythms in Ca^2+^ and cAMP levels (see Sections 1.4, 3.3). These membrane-associated oscillations at different timescales can be phase-shifted by neurotransmitters and neuropeptides that signal via G protein-coupled receptors (Ikeda et al., 2003b; Ikeda et al., 2003a; Schendzielorz et al., 2012; Schendzielorz et al., 2014; Schendzielorz et al., 2015; Wei and Stengl, 2012; Brancaccio et al., 2013; Brancaccio et al., 2017; Wei et al., 2014; Stengl et al., 2015; Stengl and Arendt, 2016; Harvey et al., 2020; Parnell et al., 2021; Stengl and Schröder, 2021; Matsuo et al., 2022). Several pathways have been identified in circadian clock neurons that demonstrate how these membrane-dependent rhythms in Ca^2+^ and cAMP levels couple to the molecular clockwork in the nucleus (Figure 5): Different Ca^2+^- and cAMP-dependent kinase pathways modify relevant transcription factors such as CREB, thereby phase-locking second messenger oscillations to the core TTFL clockwork oscillation (Harvey et al., 2020; Parnell et al., 2021). These signaling pathways connect plasma membrane-dependent activity with transcriptional control in the nucleus and are employed in many processes of homeostatic plasticity that stabilize ultradian spiking patterns of neurons (Turrigiano, 2012; Steven et al., 2020; Wu et al., 2021; Fitzpatrick and Kerschensteiner, 2023; Nieto-Felipe et al., 2023).

It is generally accepted that circadian membrane potential oscillations, which are tightly interconnected with circadian rhythms of second messenger cascades, do not drive but synchronize with the circadian TTFL clockwork. It remains to be studied whether these mechanisms of circadian synchronization/coupling are identical to general neuronal homeostatic plasticity mechanisms that work on fast and slow timescales (Turrigiano, 2008; Fox and Stryker, 2017; Liu et al., 2021; Parnell et al., 2021).

## 3 Ion channels underlying circadian membrane potential oscillations are not primarily TTFL-dependent but are hubs of homeostatic plasticity

While circadian rhythms in electrical activity were observed in mammalian and insect circadian clock neurons, here, we focus mainly on the mammalian SCN (Cao and Nitabach, 2008; Colwell, 2011; Flourakis and Allada, 2015; Smith et al., 2019; Harvey et al., 2020; Barber et al., 2021; Fernandez-Chiappe et al., 2021; Stengl and Schröder, 2021; Schellinger et al., 2022). During the day, when nocturnal mice and rats sleep, most GABAergic SCN neurons express higher spike rates, higher intracellular Ca^2+^ levels, and higher input resistance as compared to the night (Pennartz et al., 2002; Colwell, 2011; Harvey et al., 2020; Patton and Hastings, 2023; Yang et al., 2023). This correlates with a multitude of ion channels in mammalian clock neurons which express daily rhythms in their current amplitudes and/or mRNA levels (Pitts et al., 2006; Colwell, 2011; CircaDB, 2013; Harvey et al., 2020; Hermanstyne et al., 2023).

During the night, the activity phase of rodents, the spontaneous spike frequency of most GABAergic SCN circadian clock neurons is lower as compared to the day. This correlates with a lower input resistance, apparently due to an increase in open time probability of hyperpolarizing ion channels. Thus, at night, hyperpolarizing ionic currents such as BK-type Ca^2+^ activated K^+^ currents dominate as compared to the day when depolarizing currents dominate (Flourakis et al., 2015; Harvey et al., 2020).

It is generally assumed that all membrane-dependent circadian rhythms, such as daily rhythms in ionic current amplitudes, are mere outputs of the molecular master TTFL clockwork (e.g., reviews: Harvey et al., 2020; Patton and Hastings, 2023). Thus, it is assumed that the TTFL clockwork activates daily expression of hyperpolarizing ion channels at night and activates daily expression of depolarizing ion channels during the day, connected to a strict circadian control of ion channel protein turnover. However, early models have shown that circadian changes in the electrical activity of neurons can be generated and sustained by the properties of ion channels and lipid membranes alone, without requiring transcriptional control connected to temporally regulated protein turnover (Njus et al., 1974).

To determine whether the TTFL clockwork in circadian clock neurons indeed controls membrane-derived circadian rhythms it would be necessary to show that the proteins of ion channels, such as pacemaker channels which are mandatory for expression of spontaneous endogenous circadian electrical rhythmicity, show circadian turnover and that respective ion channel genes require circadian transcriptional control by the TTFL clockwork. Current experiments mostly searched for correlations in maxima of ion channel mRNA levels with peaks in circadian spike rhythms in the SCN. In addition, pharmacological experiments using ion channel agonists and antagonists interfered with observed circadian firing rhythms of clock neurons, mostly at the network level (Colwell, 2011; Harvey et al., 2020; Patton and Hastings, 2023).

### 3.1 Pacemaker ion channels in circadian clock neurons that control spontaneous activity are not under strict TTFL control

Two types of leak channels with antagonistic effects (K_2P_ potassium leak and NALCN-type sodium leak channels) that control ultradian oscillations of spontaneous activity in circadian clock neurons of mammals would be ideal targets for the circadian modulation of spontaneous membrane potential oscillations (Brown and Piggins, 2007; Colwell, 2011; Flourakis et al., 2015; Harvey et al., 2020). While the current through the hyperpolarizing K_2P_ channels does not express daily rhythms the currents through depolarizing NALCN-type (insect ortholog: NARROW ABDOMEN; NA) Na^+^ leak channels express higher amplitudes during the day. Therefore, it was suggested that under the control of the molecular TTFL clock NALCN channels are responsible for the stronger depolarization during the day (Talley et al., 2001; Cochet-Bissuel et al., 2014; Flourakis et al., 2015).

Next to the NALCN-type channels other pacemaker channels support spontaneous membrane potential oscillations in circadian clock neurons, such as T-type Ca^2+^ channels, and I_h_ -type cation channels (Akasu et al., 1993; Notomi and Shigemoto, 2004; Atkinson et al., 2011). In addition, subthreshold depolarization and oscillations are driven by TRPM4 cation channels, which are both voltage- and Ca^2+^ dependent (Li et al., 2021). Furthermore, L-type Ca^2+^ channels were found to control spontaneous activity of SCN neurons (Pennartz et al., 2002; Filosa and Putnam, 2003; Jackson et al., 2004; Imber and Putnam, 2012; Sanchez-Padilla et al., 2014). It remains to be examined which of the voltage-dependent ion channels, such as L-type Ca^2+^ channels or fast delayed rectifier (FDR) and A-type K^+^ channels, are driven secondarily by the stronger baseline depolarization in circadian clock neurons during the day, or primarily via transcriptional control (Colwell, 2011; Harvey et al., 2020).

### 3.2 TTFL and PTFL regulation of ion channels connected to homeostatic plasticity

As explained in previous sections membrane potential dynamics are connected to dynamics in the concentration of intracellular Ca^2+^, which is a central intracellular messenger in a multitude of signaling cascades connected to homeostatic control. Membrane-associated signaling cascades are often restricted to subcellular nanodomains and signalosomes to ensure specific responses (Musheshe et al., 2018; Tenner et al., 2020; Zaccolo et al., 2021; Anton et al., 2022; Lohse et al., 2023; Posner et al., 2023). Similarly, not all ion channel types are uniformly distributed throughout a neuron. Their spatial distribution influences the signaling properties of a neuron (Lai and Jan, 2006). Ion channels and their associated signaling molecules form channelosomes, which present a hub for interactions with other signalosomes to regulate ion channel activity. Since the activity of ion channels can be regulated by posttranslational modifications, such as trafficking to and localization in the plasma membrane or gating kinetics, it would be generally possible to achieve circadian and ultradian control independent of the molecular TTFL clockwork (Lamothe and Zhang, 2016).

The depolarizing NALCN is linked to many cellular rhythms and signaling cascades (Flourakis et al., 2015; Lee et al., 2019; Harvey et al., 2020; Impheng et al., 2021; Kschonsak et al., 2022). It is part of a large channelosome complex and functions as a hub for signaling cascades, with links to extracellular Ca^2+^ concentrations (Cochet-Bissuel et al., 2014; Lee et al., 2019). A multitude of signals, including G protein-coupled receptors, alternative splicing, long non-coding RNA interactions, and several posttranslational mechanisms such as methylations control NALCN functions (Flourakis et al., 2015; Lee et al., 2019). Thus, it is possible that coupling of the TTFL circadian clockwork with PTFLs that maintain homeostatic control of NALCN could be required for stable rhythmicity on the circadian timescale (Lear et al., 2013; Kang and Chen, 2022). The NALCN subunit expression is not directly under transcriptional control. However, localization of NALCN to membranes via the endoplasmatic reticulum resident protein NFL-1 is controlled by the TTFL clockwork because *nlf-1* transcripts express circadian rhythms. Knockdown of *nlf-1* transcripts in different populations of clock neurons reduced NALCN protein levels, circadian spiking rhythms, as well as morning and evening anticipation in circadian locomotor behavior (Flourakis et al., 2015).

Concerning the above-mentioned K^+^ channels, circadian expression has only been observed for the subunit that determines the fast inactivation kinetics (*Kcnma1*) of the BK-type K^+^ channels during the day. On the other hand, L-type Ca^2+^ channels and FDR K^+^ channels (*Kcnc1,2*) show circadian expression levels (Colwell, 2011; CircaDB, 2013; Harvey et al., 2020). However, when L-type Ca^2+^ channels or BK-type K^+^ channels are mutated, or the TTFL clockwork was disrupted, circadian membrane potential rhythms persist, albeit with reduced stability and amplitude (Colwell, 2011; Harvey et al., 2020). Thus, only a small fraction of ion channel mRNA level oscillations depends on the molecular circadian TTFL clockwork, and they do not appear to drive all the circadian rhythms of electrical activity in clock neurons.

The current hypothesis is that circadian rhythms in SCN spike rates are caused by an increased expression of hyperpolarizing ion channels during the night. While different K^+^ channels express higher current amplitudes at night, only deletions or knock-down of K_v_12.1 and K_v_12.2-encoded K^+^ channels (*Kcnh8*, *Kcnh2* locus from the ether-á-go-go (EAG) family of voltage-gated K^+^ channels (Bauer and Schwarz, 2018)) deleted the circadian rhythm in electrical activity (Hermanstyne et al., 2023). Interestingly, mRNA levels of both K_v_12 channels do not show any circadian rhythm, and deletions of K_v_12.1, K_v_12.2 leaves behavioral circadian rhythms intact (Hermanstyne et al., 2023). Thus, their circadian rhythm is not directly controlled by the molecular circadian TTFL clockwork. Other, so far not identified, mechanisms must drive the daily rhythms in those K_v_12-dependent current levels. Members of the EAG family are hubs for several homeostatic processes and are linked to the control of membrane excitability, intracellular pH, Ca^2+^, and cyclic nucleotide levels (Bauer and Schwarz, 2018). Therefore, it is likely that they are controlled by PTFL-based circadian oscillators/clocks that are employed in mechanisms of homeostatic plasticity (O’Leary et al., 2014). Oscillating spike rates that are based on voltage and Ca^2+^ (-dependent) oscillations are maintained via mechanisms of homeostatic plasticity that comprise, amongst others, the activity-dependent control of ion channel expression levels (e.g., Colwell, 2011; Turrigiano, 2012; Lee and Kirkwood, 2019; Harvey et al., 2020; Hickey et al., 2020; McCormick et al., 2020; Wu et al., 2021; Kschonsak et al., 2022; Brodt et al., 2023; Fitzpatrick and Kerschensteiner, 2023; Valakh et al., 2023).

In summary, more than half of all ion channels found in circadian clock neurons express daily oscillations in current amplitudes while only few of these daily rhythms are directly controlled at the level of transcription via the molecular circadian TTFL clockwork. Biochemical data concerning timing of ion channel protein turnover are missing. None of the ion channels that are shown to be directly controlled by the TTFL clockwork were proven to be a mandatory prerequisite for the circadian rhythms in spike frequency or behavioral activity. However, circadian changes in the electrical activity stabilizes the output of the molecular clockwork, and circadian output of the molecular clockwork stabilizes the circadian changes in electrical activity (Colwell, 2011; Harvey et al., 2020). Furthermore, published data are neither consistent nor conclusive because the small, abundant SCN clock neurons could not be identified individually and appear to express different combinations of ion channels. It becomes increasingly apparent that a multitude of posttranslational feedback loops (PTFL oscillators) intertwine with the TTFL clockwork which are coupled to manifold receptor-dependent signaling cascades, driven by homeostatic control mechanisms that work at different timescales (Turrigiano, 2012; Li et al., 2014; Lee and Kirkwood, 2019; Hickey et al., 2020; McCormick et al., 2020; Parnell et al., 2021; Wu et al., 2021; Kschonsak et al., 2022; Tang et al., 2022; Yang et al., 2022; Brodt et al., 2023; Fitzpatrick and Kerschensteiner, 2023; Ma et al., 2023; Valakh et al., 2023).

### 3.3 Signalosomes and nanodomains

The mechanisms of homeostatic plasticity are intimately connected to PTFL oscillators that constitute localized signaling cascades, termed signalosomes. Signalosomes are multimolecular complexes that anchor complete signal transduction cascades from the receptor to effectors at distinct cellular locations, such as to the plasma membrane, through scaffolding molecules (Dunn and Ferguson, 2015; Zaccolo et al., 2021; Excoffon et al., 2022; Ma et al., 2023; Paolocci and Zaccolo, 2023). They allow for compartmentalization of intracellular (second) messengers in subcellular nanodomains (Zaccolo et al., 2021). Furthermore, signalosomes can constitute oscillators controlling second messenger oscillations via homeostatic mechanisms.

Especially well studied is the cAMP-dependent signal transduction cascade (Blair and Baillie, 2019; Zaccolo et al., 2021; Paolocci and Zaccolo, 2023). Extracellular signal-dependent G-protein coupled receptors activate adenylyl cyclases via G_αs_ causing rises in cAMP levels, or inhibit adenylyl cyclases via G_αi_ (Rodbell et al., 1971; Tulsian et al., 2020; Boczek et al., 2021). The changing cAMP concentrations can modulate ion channels (Fesenko et al., 1985; Kar et al., 2021; Saponaro et al., 2021; Lohse et al., 2023), as well as cAMP-dependent protein kinase A (PKA) (Walsh et al., 1968; Corbin and Keely, 1977; Taylor et al., 1993; Taylor et al., 2013; Diskar et al., 2010; Wiggins et al., 2018; Paolocci and Zaccolo, 2023), cAMP exchange protein (EPAC) (de Rooij et al., 1998), or Popeye domain-containing proteins (POPDC) (Brand, 2005). Via different mechanisms PKA actions can remain localized (Smith et al., 2017; Walker-Gray et al., 2017; Chao et al., 2019). For example, PKA anchoring proteins (AKAPs) can keep activated PKA compartmentalized, as opposed to the previous assumption that the catalytic PKA subunit may diffuse freely in the cytoplasm (Colledge and Scott, 1999; Wong and Scott, 2004; Bachmann et al., 2016).

Next to the positive feedforward cascades connecting rising cAMP concentrations to the respective effectors, cAMP levels are restricted via negative feedback mechanisms such as hydrolysis via differently regulated phosphodiesterase that provide further links between different signaling cascades. Different isoforms of phosphodiesterase can be regulated directly or indirectly (e.g., via respective phosphorylations) via cAMP, Ca^2+^, and cGMP levels confined to specific subcellular locations (Marchmont and Houslay, 1980; Conti et al., 1984; Jurevicius and Fischmeister, 1996; Mongillo et al., 2004; Bender and Beavo, 2006; Lugnier, 2006; Di Benedetto et al., 2008; Weber et al., 2017; Vinogradova and Lakatta, 2021; Paolocci and Zaccolo, 2023). Furthermore, cAMP signaling can be curtailed via phosphatases that antagonize PKA-dependent phosphorylations (Ingebritsen and Cohen, 1983; Francis et al., 2011; Mehta and Zhang, 2021).

In summary, we hypothesize that plasma membrane associated PTFL clocks constitute localized signalosomes maintained via mechanisms of homeostatic plasticity, interlinked with other PTFL and TTFL clocks/oscillators via their signaling cascades as coupling factors.

## 4 Discussion: our novel hypothesis, final conclusion, and outlook

Our opinion paper focuses on membrane-associated ultradian and circadian rhythms in insect and mammalian clock neurons, offering a new perspective by taking a systemic view on biological timing. We suggest bridges between concepts of chronobiology and homeostatic or Hebbian plasticity in the neurosciences (for details please see previous sections with respective citations). We present a fresh view of the plasma membrane as an endogenous plastic multiscale PTFL clock, tightly coupled to multiscale molecular TTFL clocks. Interlinked posttranslational feedback loops as part of PTFL clocks guarantee a balance of physiological homeostasis and plasticity at all timescales that are relevant for dynamic brain function (Figure 5). The various elements of these PTFL and TTFL oscillators/clocks are suggested to have multiple systemic functions as in- and outputs, as gates and coupling signals, as functional units in mechanisms of homeostatic or Hebbian plasticity.

How dynamic setpoints of homeostasis in brain function are controlled is one of the most important tasks to be resolved in brain research with imminent relevance in medicine and healthcare. Based on our own work as electrophysiologists experienced with individually identifiable invertebrate clock neurons (Stengl et al., 2015; Stengl and Arendt, 2016; Stengl and Schröder, 2021) we suggest a paradigm switch, presenting our novel hypothesis together with an outlook comprising suggestions how to challenge our hypothesis experimentally.

### 4.1 Our hypothesis: the plasma membrane forms a system of plastic, multiscale PTFL-based endogenous oscillators/clocks comprising different signalosomes, linked to external zeitgebers and intracellular oscillators

Based on our electrophysiological analysis of insect circadian clock neurons (reviews: Stengl, 2010; Stengl and Arendt, 2016; Stengl and Schröder, 2021) we propose that the excitable plasma membrane of spontaneously active clock neurons contains a system of coupled, endogenous, adaptive PTFL oscillators/clocks, each generating membrane potential oscillations at a characteristic infradian, ultradian, or circadian oscillation frequency (Figure 5). At the core of each neuronal membrane oscillator/clock, associated with signalosomes, specific pacemaker channels drive specific frequencies of membrane potential oscillations. The channels’ activity is tightly coupled to oscillations in concentrations of specific intracellular messengers like Ca^2+^ or cAMP (Craven and Zagotta, 2006; Biel and Michalakis, 2009; Johnstone et al., 2018; Stengl and Schröder, 2021). The Ca^2+^ oscillations in turn interlink with endogenous cAMP oscillations, e.g., via Ca^2+^-dependent adenylyl cyclases, or *vice versa* via cAMP-dependent Ca^2+^ permeable ion channels (Biel and Michalakis, 2009; Islam, 2020; Schultz, 2022; Li et al., 2023a). Via different intracellular messenger cascades multiscale membrane-associated oscillations link to multiscale TTFL oscillators/clocks engaged in gene regulation networks (Colwell, 2011; Patton et al., 2016; Stengl and Arendt, 2016; Hastings et al., 2019; Perfitt et al., 2020; Steven et al., 2020; Stengl and Schröder, 2021). The endogenous oscillations in membrane potential and second messenger concentrations are coupled/entrained via various receptors which repetitively phase-shift the respective endogenous oscillations until they remain synchronized with the respective internal oscillator or external zeitgeber.

We suggest that signalosomes associated with the plasma membrane of sensory clock neurons in different parts of the body comprise receptors specialized for the detection of specific environmental rhythms/zeitgebers such as the daily light dark cycle or fast chemical fluctuations (Calebiro and Grimes, 2020; Erofeeva et al., 2023; Takeuchi and Kurahashi, 2023). Signalosome receptors of central clock neurons in the brain link to other physiological oscillators/clocks via neurotransmitters, neurohormones, and neuropeptides as coupling factors (Choi et al., 2012; Wei et al., 2014; Gestrich et al., 2018; Ono et al., 2021). Depending on the signalosome-specific receptors and signal transduction cascades, entrained rhythms of specific intracellular messenger concentrations are generated that oscillate at the same frequency as their respective zeitgeber or coupled oscillator/clock forming an interlinked system of physiological/behavioral timing (Figure 5).

We hypothesize that the negative feedback elements of the membrane associated PTFL oscillators are identical to mechanisms of homeostatic plasticity that act as gain control mechanisms preventing runaway activations. For example, in hawkmoth pheromone-receptor neurons, which are both ultradian and circadian oscillators, Ca^2+^ activates non-specific cation channels that also permeate Ca^2+^. The influx of Ca^2+^ via the cation channel then decreases their open-time probability if intracellular Ca^2+^-concentrations rise above a certain level (Stengl, 1993; Stengl and Schröder, 2021). Negative feedback mechanisms are suggested to maintain respective dynamic setpoints (i.e., stable oscillation frequency). Alternatively, via different forms of non-associative learning (adaptation, sensitization) or associative forms of learning (Hebbian learning) new dynamic setpoints at different frequencies can be obtained (as described in previous sections).

In contrast to a hierarchical view of TTFL clocks that generate all rhythms via transcriptional control we suggest that specific TTFL- or PTFL-based clocks/oscillators can become dominant or dormant, dependent on the respectively impacting zeitgeber/coupling factor, or respective physiological or behavioral circumstances. Furthermore, we suggest that zeitgeber-dependent oscillations in intracellular concentrations of Ca^2+^ and cAMP, and the type and relative amounts of activated kinases and phosphatases, play a key role as coupling factors. They couple oscillations at different timescales, couple PTFL- and TTFL clockworks in single clock neurons, and connect various nanodomains via controlled diffusion (Wei et al., 2014). For example, PKC and/or PKA can act as coupling factors between PTFL- and TTFL clockworks by modification of CREB or other transcription factors that are positive feedforward elements of TTFLs (Colwell, 2011; Patton et al., 2016; Stengl and Arendt, 2016; Hastings et al., 2019; Perfitt et al., 2020; Steven et al., 2020; Stengl and Schröder, 2021).

Furthermore, at the network level signalosomes may comprise various types of neuropeptide- and neurotransmitter receptors to mediate coupling between clock neurons. Neuropeptides or neurotransmitters could act as coupling factors or as gates that reprogram a neuronal circuit via phase-dependent synchronization of receptor expressing neurons in the network (Schneider and Stengl, 2005). Thus, neuropeptides cause neuronal ensemble formation and thereby change homeostatic dynamic setpoints in neuronal networks, guiding the brain to a new physiological and behavioral context (Marder, 2012; Stengl et al., 2015; Stengl and Arendt, 2016).

### 4.2 Conclusion

We suggest that a stably coupled, interconnected system of endogenous oscillators and clocks oscillating at defined frequency bands defines dynamic homeostatic setpoints of physiology and behavior. This systems-based concept comprising different time scales and different levels of complexity differs from the currently dominating hierarchical concepts of chronobiology (e.g., Harvey et al., 2020; Rosbash, 2021; Patton and Hastings, 2023) but appears to find increasing support (Hall, 2005; Stengl et al., 2015; Stengl and Arendt, 2016; Rojas et al., 2019; Jaumouillé et al., 2021; Stengl and Schröder, 2021; Yang et al., 2022; Heigwer et al., 2023). Furthermore, contrasting the current hypothesis (Hughes et al., 2009; Zhu et al., 2017; Ballance and Zhu, 2021) we propose an equally important role for plasma membrane associated PTFL clocks as compared to TTFL clocks associated with nuclear gene regulatory networks. The PTFL membrane clocks generate superimposed endogenous multiscale oscillations in membrane potential and intracellular messenger levels connecting to multiscale TTFL oscillations. Like waves on the surface of a lake, endogenous ultradian, circadian, and infradian oscillations of the membrane potential spread from their locally structured signalosomes across the plasma membrane to other compartments of the clock cells. The stable systemic superposition of oscillations at different timescales, originating at diverse signalosomes are suggested to allow equally for stability and plasticity, being hallmarks of the neuron’s dynamic homeostatic setpoints (Figure 5) (Hutcheon and Yarom, 2000; Hughes et al., 2009; Albert, 2011; Cochet-Bissuel et al., 2014; Ananthasubramaniam et al., 2018; Bauer and Schwarz, 2018; Alza et al., 2022; Hille, 2022; Bronson and Kalluri, 2023).

### 4.3 Outlook

To challenge our hypothesis and to reveal mechanisms of timing that span multiple timescales, it would be necessary to perform long-term physiological experiments with endogenous clock neurons over several days. With improved data analysis and modelling a careful search for periodicities at different timescales, that can be detected as stably oscillating frequency band, needs to be performed, with/without compromising the TTFL clockwork. Furthermore, since clock neurons in the SCN or the insect AME clock are very heterogeneous it is necessary to work with individually identified and characterized endogenous clock neurons over the course of the respectively expressed endogenous ultradian and circadian cycles. Thus, long-term physiological recordings of primary cell cultures *in vitro* and of intact clock networks *in situ* need to be accomplished, such as cell-attached patch clamp recordings in search for oscillations in electrical activity at specific interlinked frequency bands, as candidates for “physiological fingerprints”. In search of interlinked intracellular messenger oscillations long-term imaging of Ca^2+^and/or cAMP levels in individual clock cells needs to be performed together with electrophysiological analysis. These experimentally very challenging experiments will reveal and characterize specific physiological clock types. Single-cell transcriptomics and single cell mass spectrometry at different times of the circadian and ultradian cycles will reveal different types of clock neurons at the transcriptional level and will identify candidates of coupling factors. With refined data analysis and modelling the different types of clock cells need to be matched and their interdependence in the network needs to be calculated. Furthermore, most interesting is to determine which modification of predicted coupling factors affect physiological oscillations at different timescales as predicted hallmarks of dynamic setpoints of homeostasis and plasticity. Finally, combined physiological and behavioral assays need to determine whether changes of the respective oscillation frequencies (“physiological fingerprints”) correlate with/are functionally connected to changes in dynamic homeostatic setpoints. Currently, we are testing our hypothesis in respective long-term assays of peripheral and central insect circadian clock cells in the hawkmoth *Manduca sexta* and the cockroaches *Rhyparobia maderae* and *Periplaneta americana.* We encourage the interested scientists to try to falsify our new hypothesis experimentally in their respective model systems.

## Data Availability

The original contributions presented in the study are included in the article, further inquiries can be directed to the corresponding author.
